# ﻿Taxonomic notes on the crab-spider genera *Nyctimus* Thorell, 1877 and *Zametopina* Simon, 1909 (Araneae, Thomisidae) with descriptions of six new species from Southeast Asia

**DOI:** 10.3897/zookeys.1255.158380

**Published:** 2025-10-09

**Authors:** Suresh P. Benjamin, Naufal Urfi Dhiya’ulhaq, Christa Deeleman-Reinhold, Damayanti Buchori, Purnama Hidayat, Stefan Scheu, Jochen Drescher

**Affiliations:** 1 National Institute of Fundamental Studies, Hantana Road, Kandy 20000, Sri Lanka; 2 Animal Ecology, J.F. Blumenbach Institute of Zoology and Anthropology, University of Göttingen, Untere Karspüle 2, Göttingen 37073, Germany; 3 Species Obscura, Jl. Duren 1 No.2, Depok, Jawa Barat 16434, Indonesia; 4 Netherlands; 5 Department of Plant Protection, Faculty of Agriculture, IPB University, Kampus IPB Dramaga Bogor, Jl. Raya Dramaga, West Java 16680, Indonesia; 6 Centre for Transdisciplinary and Sustainability Sciences, IPB University, Kampus IPB Baranangsiang, Jl. Raya Pajajaran No.27, West Java 16127, Indonesia; 7 Centre of Biodiversity and Sustainable Land Use, University of Göttingen, Büsgenweg 1, Göttingen 37077, Germany; 8 Senckenberg Museum for Natural Sciences Görlitz, Am Museum 1, 02826 Görlitz, Germany; † Deceased

**Keywords:** Arachnida, biodiversity, RTA-clade, taxonomy

## Abstract

The crab-spider genera *Zametopina* Simon, 1909 and *Nyctimus* Thorell, 1877 remain poorly studied, with significant gaps in their taxonomic understanding. *Nyctimus* has seen little study since its original description, and species assignments remain uncertain due to a lack of modern diagnostic data. By contrast, *Zametopina*, historically monotypic, was recently expanded with a second species (*Z. wanliae* Lin & Li, 2023) following a redescription of the type species, *Z. calceata* Simon, 1909. Our study presents a review of these genera, primarily based on specimens collected from tropical Asia by the late Christa L. Deeleman-Reinhold as well as from the EFForTS project in the lowlands of Sumatra, Indonesia. The review includes the description of the following six new species: *Nyctimus falcatus* Benjamin & Dhiya’ulhaq, **sp. nov.** (♂), *Nyctimus kinabaluensis* Benjamin & Dhiya’ulhaq, **sp. nov.** (♂♀), *Nyctimus mutilloides* Benjamin & Dhiya’ulhaq, **sp. nov.** (♂), *Nyctimus quadripunctatus* Dhiya’ulhaq & Benjamin, **sp. nov.** (♂♀), *Nyctimus rendang* Dhiya’ulhaq & Benjamin, **sp. nov.** (♂♀), and *Nyctimus saksang* Dhiya’ulhaq & Benjamin, **sp. nov.** (♂). *Nyctimus bistriatus* Thorell, 1877 is redescribed and new locality records are provided. *Zametopina wanliae* is recorded for the first time outside of Vietnam. Further, updated species descriptions of *Nyctimus* and *Zametopina* are provided, along with taxonomic clarifications of the genera. This study emphasises the fundamental importance of well-curated, historical collections and ongoing fieldwork in resolving the systematics of lesser-known arachnid lineages.

## ﻿Introduction

*Zametopina* Simon, 1909, is a genus of crab spiders belonging to the family Thomisidae. Referred to as crab spiders, due to their elongated first two leg pairs and their habit of moving sideways, Thomisidae are among the most species-rich spider families with more than 2,100 species across 171 genera (World Spider Catalog 2025). Despite its inclusion in this diverse group, *Zametopina* remains relatively obscure and taxonomically unrevised. Initially, the genus comprised only a single species, *Zametopina calceata* Simon, 1909, which had been inadequately understood for decades due to a lack of illustrations in the original description. A significant update came with the work of Tang et al. (2010), who revisited and redescribed the type species. Through meticulous examination of the type specimen, they provided detailed illustrations and descriptions, thereby clarifying the diagnostic features of the genus and aiding in its taxonomic circumscription. This effort significantly enhanced the understanding of *Zametopina* and set the stage for future taxonomic studies. Recently, a second species, *Zametopina wanliae* Lin & Li, 2023, was described in Lin et al. (2023), further enriching the diversity of the genus.

By contrast, the genus *Nyctimus* Thorell, 1877, has received limited taxonomic scrutiny since its initial description. Historically, *Nyctimus* included two species: the type species *Nyctimus bistriatus* Thorell, 1877, from Indonesia (Sumatra and Sulawesi), and *Nyctimus trimeni* (Simon, 1895) from South Afri­ca. Originally described as *Zametopias trimeni*, the latter is likely misplaced, indicating a need for taxonomic revision. Additionally, *Zametopias speculator* Thorell, 1892, was transferred to *Nyctimus* and synonymised with *N. bistriatus* by Lehtinen (2016). However, this transfer was made without accompanying illustrations or a comprehensive re-examination of the type material, leaving the taxonomy of both species inadequately resolved.

This paper aims to address some of these gaps by reviewing the poorly known species of both *Zametopina* and *Nyctimus*, primarily drawing on specimens collected from tropical Asia by the late Christa L. Deeleman-Reinhold, as well as from the EFForTS project in the lowlands of Jambi Province, Sumatra, Indonesia (Drescher et al. 2016). This review aims to clarify the taxonomy and provide updated descriptions, contributing to a more robust understanding of these genera and their roles in the biodiversity of the regions they inhabit. The study highlights the importance of revisiting historical collections and underscores the need for continued exploration and documentation of lesser-known spider taxa.

## ﻿Materials and methods

### ﻿Sample collection

Specimens from the late Christa L. Deeleman-Reinhold’s personal collection, housed at her residence, were selected by Suresh P. Benjamin (SPB) and transferred to the Zoological Research Museum Alexander König (Bonn, Germany, ZFMK) for this study. These specimens, along with the rest of her collection, will ultimately be deposited at Naturalis Biodiversity Center, Leiden, Netherlands (RMNH). Spiders from Jambi Province were part of a collection of more than half a million canopy arthropods (Pollierer et al. 2023) sampled by canopy fogging across rainforest, jungle rubber, and monocultures of rubber and oil palm in the lowlands of Jambi, Sumatra, Indonesia as part of the German-Indonesian EFForTS research project (Drescher et al. 2016). Overall, more than 10,000 spider individuals from at least 30 families and 400 species were captured (Ramos et al. 2022; Dhiya’ulhaq et al. 2024). The fogging method is detailed in Pollierer et al. (2023) and the EFForTS plot design and general study aims in Drescher et al. (2016).

### ﻿Identification and photography

The general methodology follows Benjamin (2011) and Ileperuma Arachchi and Benjamin (2019). Specimens used for habitus illustrations were placed in 70% EtOH and photographed with a Zeiss AxioCam HRc camera mounted on a dissecting microscope (Zeiss Discovery V20) with top illumination and a magnification of up to 150×. Images were edited using the Zeiss ZEN Pro software package. Unless otherwise stated, depicted structures are of the left male palp. Alternatively, some specimens were examined under a ZEISS Stemi 2000 microscope. Female genitalia were excised from the specimen’s body and then cleared with 10% KOH for at least one hour to examine the internal copulatory organs. Imaging the specimens was done using a Keyence VHX-7000 digital microscope system or a Zeiss AxioCam HRc camera mounted on a dissecting microscope (Zeiss Discovery V20). The description of colouration is based on specimens in ethanol. Measurements of legs are given as total length (femur, patella, tibia, metatarsus, tarsus). Missing legs or leg segments are marked with “-”, and legs with missing segments do not have their total length recorded. Unmentioned legs did not have their length measured. All measurements are in millimeter.

### ﻿Abbreviations

**ALE** anterior lateral eye

**AME** anterior median eye

**CO** copulatory opening

**FD** fertilization duct

**PLE** posterior lateral eye

**PME** posterior median eye

**RTA** retrolateral tibial apophysis of male palp

**VTA** ventral tibial apophysis of male palp

### ﻿Repositories

**GOET**Animal Ecology Group, J.-F. Blumenbach Institute of Zoology and Anthropology, University of Göttingen, Göttingen, Germany (Jochen Drescher, Stefan Scheu)

**MCSN**Museo Civico di Storia Naturale Giacomo Doria, Genoa, Italy (Maria Tavano)

**MCZ**Museum of Comparative Zoology, Massachusetts (Gonzalo Giribet)

**MNHN**Muséum National d’Histoire Naturelle, Paris (Christine Rollard)

**MZB**Museum Zoologicum Bogoriense, Bogor, Indonesia (Cahyo Rahmadi)

**RMNH**Naturalis Biodiversity Center, Leiden (Karen van Dorp)

**SMF**Naturmuseum Senckenberg, Frankfurt a. M., Germany (Jana Grüger, Peter Jäger)

**ZMH**Zoologisches Museum Hamburg, Hamburg, Germany (Nadine Dupérré, Danilo Harms)

## ﻿Results

### ﻿Family Thomisidae Sundevall, 1833

#### ﻿ Nyctimus

Taxon classificationAnimaliaAraneaeThomisidae

Thorell, 1877

10DC5F15-1D15-5FB7-AE1F-EDF552C40744

##### Type species.

*Nyctimus bistriatus* Thorell, 1877.

##### Diagnosis.

**Figure 1.**
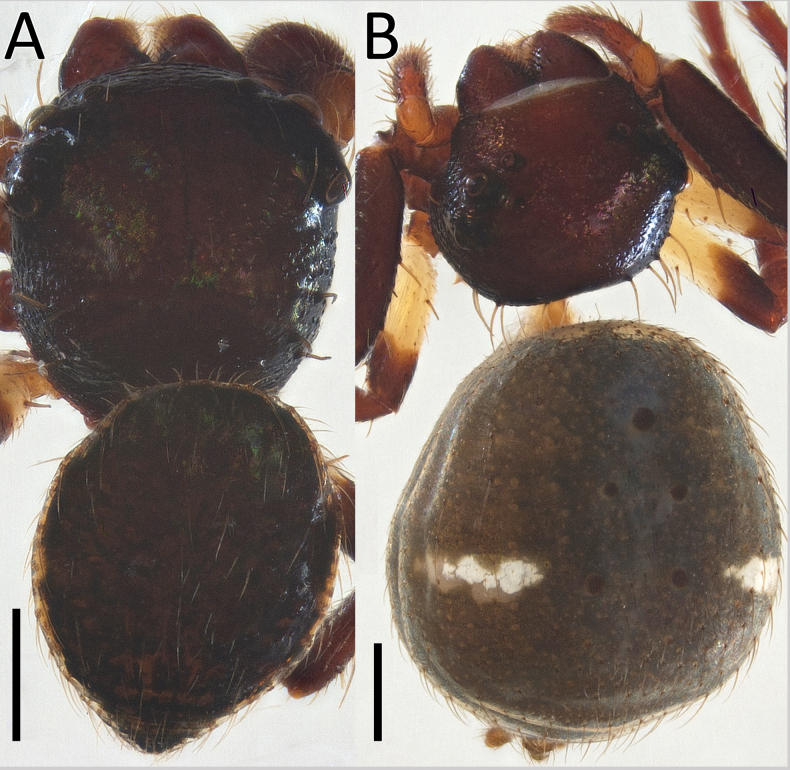
*Nyctimus bistriatus* Thorell, 1877 from Borneo; habitus, dorsal view. A. Male (RMNH.ARA.17873); B. Female (RMNH.ARA.17875). Scale bars: 0.5 mm.

Small thomisids, 2–5 mm in body length. Prosoma cuboid, as long as wide, with granulated surface and bearing long macrosetae (Fig. 1). Body colouration dark with white/yellow markings, mostly in the form of spots and bars. Opisthosoma with five large sigillae arranged in a triangular formation Embolus is weakly curved and possesses a thick base. Tegulum with a “hood” (Fig. 3A). Tibial apophyses are well developed, VTA present, either free (Fig. 3A) or hook-shaped and attached to RTA (Fig. 7A). Epigynum highly variable, spermatheca rounded or oval, single pair (Fig. 3C, D).

##### Species composition.

*Nyctimus bistriatus* Thorell, 1877 (♂♀), *Nyctimus falcatus* Benjamin & Dhiya’ulhaq, sp. nov. (♂), *Nyctimus kinabaluensis* Benjamin & Dhiya’ulhaq, sp. nov. (♂♀), *Nyctimus mutilloides* Benjamin & Dhiya’ulhaq, sp. nov. (♂), *Nyctimus quadripunctatus* Dhiya’ulhaq & Benjamin, sp. nov. (♂♀), *Nyctimus rendang* Dhiya’ulhaq & Benjamin, sp. nov. (♂♀), *Nyctimus saksang* Dhiya’ulhaq & Benjamin, sp. nov. (♂), *Nyctimus trimeni* (Simon, 1895).

#### ﻿ Nyctimus bistriatus

Taxon classificationAnimaliaAraneaeThomisidae

Thorell, 1877

632872F7-93A8-58A6-A18B-482B3EEB3773

Figs 1, 2, 3

Nyctimus bistriatus Thorell, 1877: 499; Lehtinen 2016: 166 (as Nyctimus binotatus, lapsus).
Zametopias speculator Thorell, 1892: 123.

##### Type material.

***Holotype*.** Indonesia – Sulawesi, Southeast Sulawesi Province • ♂; Kendari; 1874; O. Beccarii leg.; holotype of *Nyctimus bistriatus* Thorell, 1877; MCSN. Examined.

##### Other material examined.

Brunei – Tutong District • 1♂; Bukit Sulang; 1982; N. Stork leg.; canopy fogging in primary forest; RMNHRMNH.ARA.17873. Indonesia – Sumatra, Jambi Province • 1♀; Sarolangun, Bukit Duabelas National Park; 01°58'55.2"S, 102°45'02.6"E; elev. 73 m; 7 Oct. 2013; J. Drescher leg.; canopy fogging in rainforest; GOET 2013_BF2.1_AraThom051N_001 (to be transferred to MZB). Malaysia – Sabah State • 1♂; Kinabalu National Park, Poring Hot Springs; 05°59'N, 116°42'E; elev. 600–700 m; 27 Feb. 1996; A. Floren leg.; canopy fogging on *Aporosa lagenocarpa* tree, in primary forest; RMNHRMNH.ARA.17874. • 1♀; Kinabalu National Park, Poring Hot Springs; 05°59'N, 116°42'E; elev. 600–700 m; 28 Mar. 1998; A. Floren leg.; canopy fogging on *Barringtonia* tree, in primary forest; RMNHRMNH.ARA.17875. • 1♂; Kinabalu National Park, Poring Hot Springs; 05°59'N, 116°42'E; elev. 600–700 m; 28 Mar. 1998; A. Floren leg.; canopy fogging on *Dacryodes laxa* tree, in primary forest; RMNHRMNH.ARA.17876.

##### Diagnosis.

**Figure 2.**
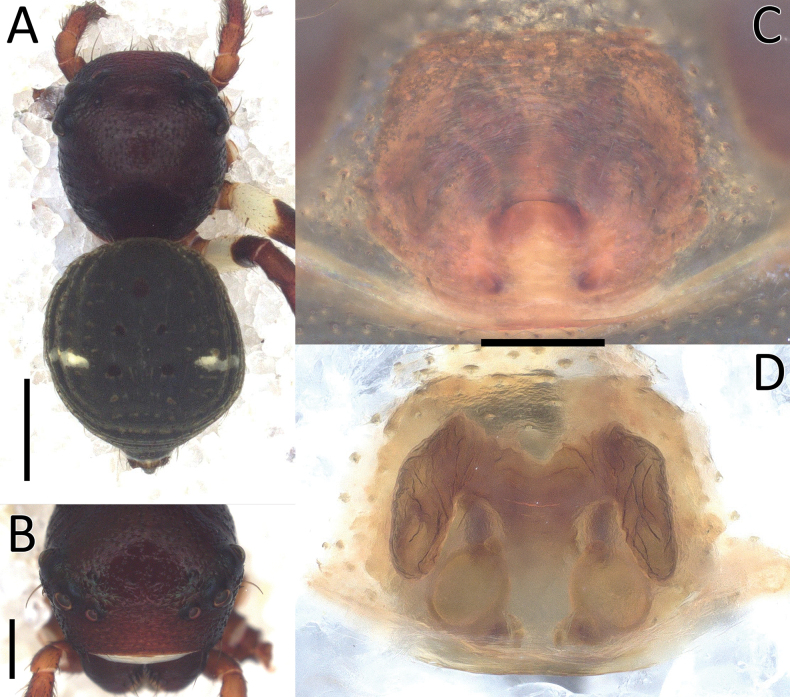
*Nyctimus bistriatus* Thorell, 1877 female from Sumatra (2013 BF2.1 AraThom051N 001). A. Habitus, dorsal view; B. Prosoma, frontal view; C. Epigynum, ventral view; D. Epigynum, dorsal view, cleared. Scale bars: 1 mm (A); 0.5 mm (B); 0.2 mm (C, D).

**Figure 3.**
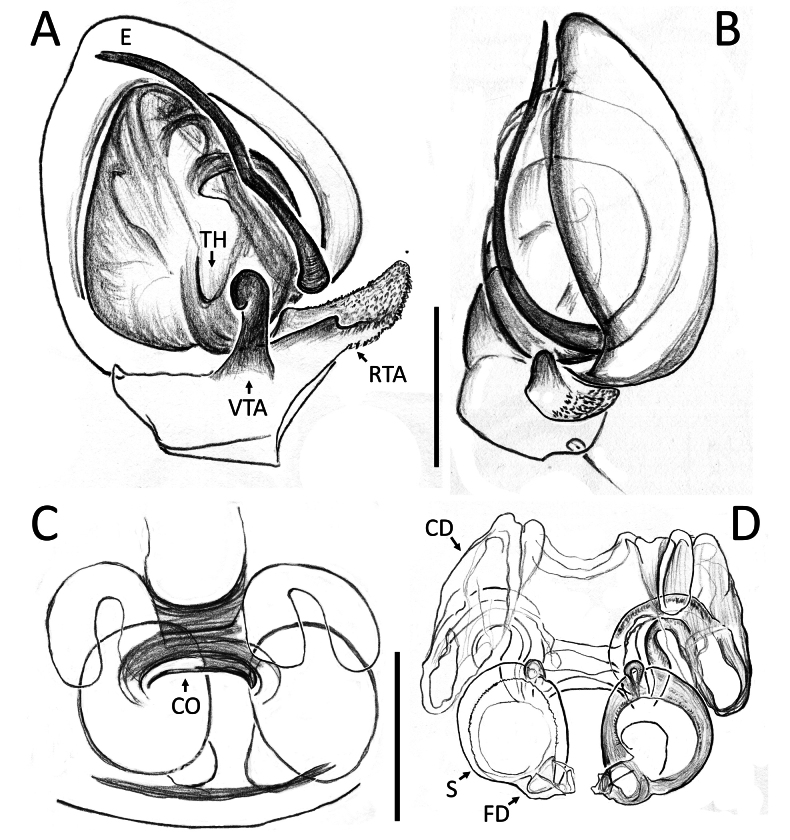
*Nyctimus bistriatus* Thorell, 1877 from Borneo. A. Male (RMNH.ARA.17873), left palp, ventral view; B. ibid., retrolateral view; C. Female (RMNH.ARA.17875), epigynum; C. ibid., ventral view; D. ibid., dorsal view. Abbreviations: CD = copulatory duct; CO = copulatory opening; E = embolus; FD = fertilization duct; RTA = retrolateral tibial apophysis; S = spermatheca; TH = tegular hood; VTA = ventral tibial apophysis. Scale bars: 0.2 mm.

Males can be easily distinguished from all other congeners by the longer embolus, more than length of cymbium (in all congeners, the embolus is at most half the length of cymbium; Fig. 3A, B) and the denticulate RTA. Females can be easily distinguished by the single CO; CD arched, resembling a rams’ horn, more than twice the length of spermathecae (in all congeners, the CO is paired, and the CD are not longer than spermathecae; Figs 2C, D, 3C, D).

##### Description.

**Male** (RMNH.ARA.17873; Figs 1A, 3A, B). Total length 2.85. Prosoma length 1.42; width 1.26. Opisthosoma length 1.43; width 1.17. Diameter of eyes: AME 0.09; ALE 0.15; PLE 0.14 PME 0.07. Interdistances between eyes: AME–AME 0.50; AME–ALE 0.10; ALE–ALE 0.86; PME–PME 0.70; PME–PLE 0.20; ALE–PLE 0.18; AME–PME 0.13; PLE–PLE 1.09. Leg measurements: leg I 3.3 (1.0, 0.5, 1.0, 0.8, 0.5). Prosoma cuboid, almost as long as wide; dorsally blackish brown, surface granulated; laterally with two rows of long, curved macrosetae originating on small tubercles. All eyes are very far apart, especially between the median eyes. Chelicerae, labium and sternum blackish brown; sternum with small dents. Legs dark brown except femora III and IV, which are yellow; femora and tibiae I–IV with two long spines; patellae I–IV with one distal long spine. Opisthosoma dorsally greyish black with many long setae; five large sigillae present, arranged in a triangular formation; venter greyish black.

Palp (Fig. 3A, B): cymbium oval. Embolus long, whip-like, looping over the retro­lateral side of bulb, half of its length covered by the cymbium. Tegular hood small, located close to the base of bulb. RTA large, roughly trapezoid, laterally protruding, distal half with denticles on all sides. VTA long, digitiform; apically flexed with tip ventrally curved.

**Female** (RMNH.ARA.17875; Figs 1B, 3C, D). Total length 3.32. Prosoma length 1.15; width 1.30. Opisthosoma length 2.17; width 1.95. Diameter of eyes: AME 0.08; ALE 0.13; PLE 0.16; PME 0.06. Interdistances between eyes: AME–AME 0.56; AME–ALE 0.10; ALE–ALE 0.86; PME–PME 0.64; PME–PLE 0.21; ALE–PLE 0.18; AME–PME 0.16; PLE–PLE 1.09. Leg measurements: leg I 5.3 (1.6, 0.6, 1.3, 1.0, 0.8). Habitus as in male, except opisthosoma dorsally lighter than male and the presence of white spots as shown in Fig. 1B.

Epigynum (Figs 2C, D, 3C, D): exterior plate roughly pentagonal. Atrium inconspicuous. CO oval, single, large, CD long; divided into a very wide, ramshorn-shaped initial part and a narrow, C-shaped later part. Spermatheca globular, approximately as wide as CO; anteriorly with a small bump; posteriorly extended.

##### Distribution.

Brunei, Malaysia (Borneo: Sabah), Indonesia (Sumatra: Jambi; Sulawesi: Southeast Sulawesi) (Fig. 19).

#### ﻿ Nyctimus falcatus

Taxon classificationAnimaliaAraneaeThomisidae

Benjamin & Dhiya’ulhaq sp. nov.

10A23D92-4157-560C-B4FD-69AF2B3C67A8

https://zoobank.org/8DF08156-9B26-4343-95A5-641924B7AC50

Fig. 4

##### Etymology.

The species epithet *falcatus* is derived from the Latin word *falx*/*falcis*, meaning “sickle” or “scythe.” It refers to the distinctly curved, sickle-shaped embolus of the male palp, a diagnostic feature of the species.

##### Type material.

***Holotype*.** Thailand – Phang Nga Province • ♂; Khao Lak National Park; no more label data; RMNHRMNH.ARA.17861.

##### Diagnosis.

**Figure 4.**
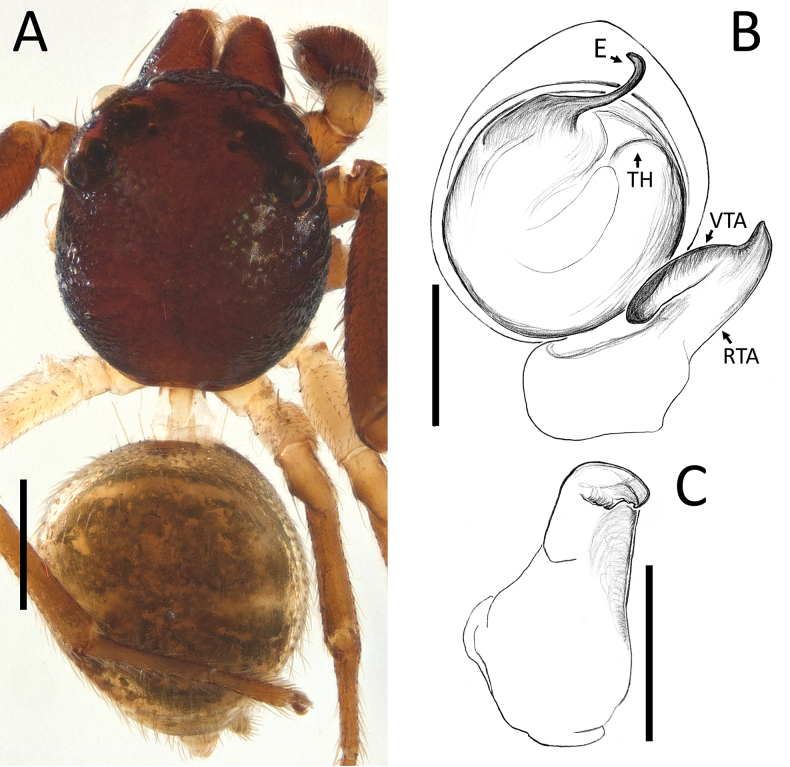
*Nyctimus falcatus* Benjamin & Dhiya’ulhaq, sp. nov., male holotype (RMNH.ARA.17861). A. Habitus, dorsal view; B. Left palp, ventral view; C. Left palpal tibia, retrolateral view. Abbreviations: E = embolus; RTA = retrolateral tibial apophysis; TH = tegular hood; VTA = ventral tibial apophysis. Scale bars: 0.5 mm (A); 0.2 mm (B, C).

Males are very similar to *N. saksang* sp. nov., in having a similar sickle-shaped embolus, but they can be easily distinguished from it by the large, digitiform VTA, attached distally on the RTA (the corresponding part in *N. saksang*, sp. nov., is much smaller and attached medially on the ventral lobe of RTA; Fig. 4B vs Figs 16C, 17A).

##### Description.

**Male** (holotype RMNH.ARA.17861; Fig. 4). Total length: 2.36. Prosoma length 1.23; width: 1.05. Opisthosoma length 1.13; width 1.02. Diameter of eyes: AME 0.08; ALE 0.15; PLE 0.14; PME 0.05. Interdistances between eyes: AME–AME 0.27; AME–ALE 0.08; ALE–ALE 0.59; PME–PME 026; PME–PLE 0.27; ALE–PLE 0.18; AME–PME 0.14; PLE–PLE 0.79. Prosoma cuboid, almost as long as wide, dark reddish brown; surface weakly granulated; laterally adorned with long macrosetae, each arising from a small tubercle. Chelicerae and sternum coloured as prosoma. Front legs brown; back legs brown except for the white femora III and IV. Opisthosoma round, light brown; dorsally covered with scutum; five large sigillae present, arranged in a triangular formation.

Palp (Fig. 4B, C): cymbium oval. Embolus sickle-shaped. Tegulum round, with a small triangular projection next to the embolus; tegular hood small. RTA wide trapezoid; VTA large, digitiform, attached distally on the RTA.

**Female.** Unknown.

##### Distribution.

Only known from the type locality (Thailand: Phang Nga) (Fig. 19).

##### Remarks.

The specimen is partially damaged: both first legs are broken and incomplete, and the remaining legs are mostly missing or fragmented.

#### ﻿ Nyctimus kinabaluensis

Taxon classificationAnimaliaAraneaeThomisidae

Benjamin & Dhiya’ulhaq sp. nov.

A719868B-03C7-5999-BD87-2DD47B99BD6E

https://zoobank.org/1F2D858B-2FED-4397-BE73-9B841300D782

Figs 5, 6, 7

##### Etymology.

The specific epithet is derived from the name of the type locality.

##### Type material.

***Holotype*.** Malaysia – Sabah State • ♂; Kinabalu National Park, Poring Hot Springs; 05°59'N, 116°42'E; elev. 600–700 m; 27 Feb. 1996; A. Floren leg.; canopy fogging on *Barringtonia* tree, in primary forest; RMNHRMNH.ARA.17862.

##### Other material examined.

Brunei – Tutong District • 1♀; Lamunin, Bukit Sulang; 1982; N. Stork leg.; canopy fogging; RMNHRMNH.ARA.17859. Indonesia – Sumatra, Jambi Province • 1♀; Sarolangun, Bukit Duabelas National Park; 01°58'55.2"S, 102°45'02.6"E; elev. 73 m; 7 Oct. 2013; J. Drescher leg.; canopy fogging in rainforest; GOET 2013_BF2.1_AraThom004N_001 (to be transferred to MZB).

##### Diagnosis.

Males can be easily distinguished from all other congeners by the embolic base abruptly bent 90° towards the retrolateral side of the cymbium and the tapered, bent embolus (Fig. 7A, B). Females are distinguishable by the long, oval spermatheca (globular or short-oval in all other congeners; Figs 6C, D, 7C, D).

##### Description.

**Figure 5.**
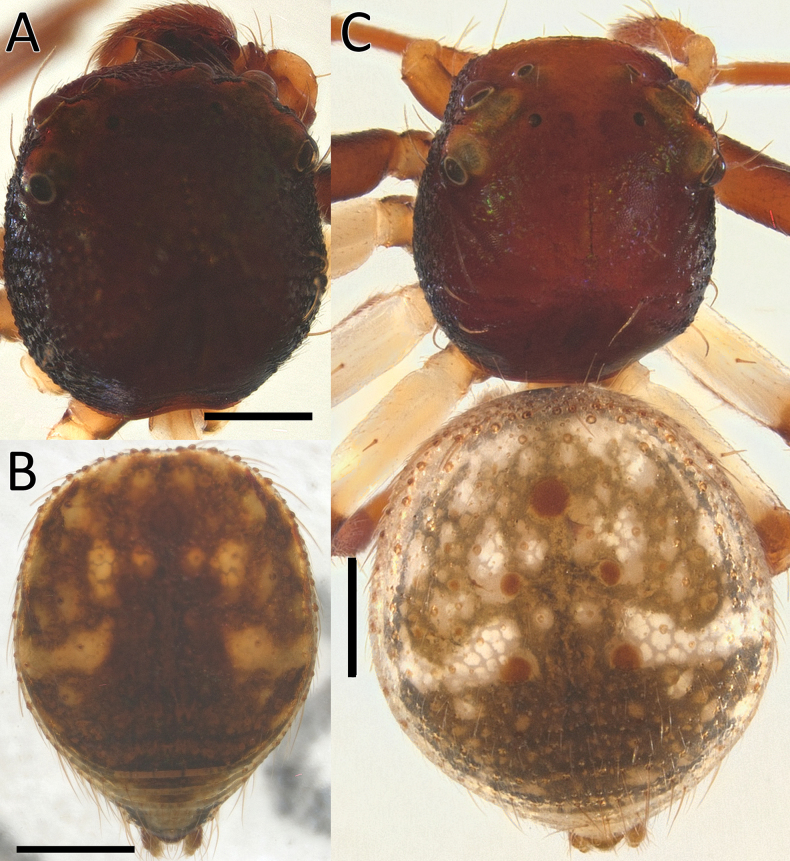
*Nyctimus kinabaluensis* Benjamin & Dhiya’ulhaq sp. nov., from Borneo. A. Male holotype (RMNH.ARA.17862), prosoma, dorsal view; B. ibid., opisthosoma, dorsal view; C. Female (RMNH.ARA.17859), habitus, dorsal view. Scale bars: 0.5 mm.

**Male** (holotype RMNH.ARA.17862; Figs 5A, B, 7A, B). Total length 3.24. Prosoma length 1.61; width 1.50. Opisthosoma length 1.63; width 1.31. Diameter of eyes: AME 0.11; ALE 0.20; PLE 0.17; PME 0.08. Interdistances between eyes: AME–AME 0.44; AME–ALE 0.11; ALE–ALE 0.81; PME–PME 0.45; PME–PLE 0.36; ALE–PLE 0.21; AME–PME 0.17; PLE–PLE 1.15. Leg measurements: leg I 5.0 (1.5, 0.6, 1.3, 1.0, 0.6). Prosoma cuboid, almost as long as wide, dark reddish brown; surface weakly granulated; laterally adorned with long macrosetae, each arising from a small tubercle. Chelicerae and sternum coloured as prosoma. Endites brown with white tip. Front legs brown; back legs brown except for the white femora. Opisthosoma oval, greyish brown with white spots (most dense in the anterior half) and paired white bars in the middle; anteriorly with a white border; five large sigillae present, arranged in a triangular formation.

**Figure 6.**
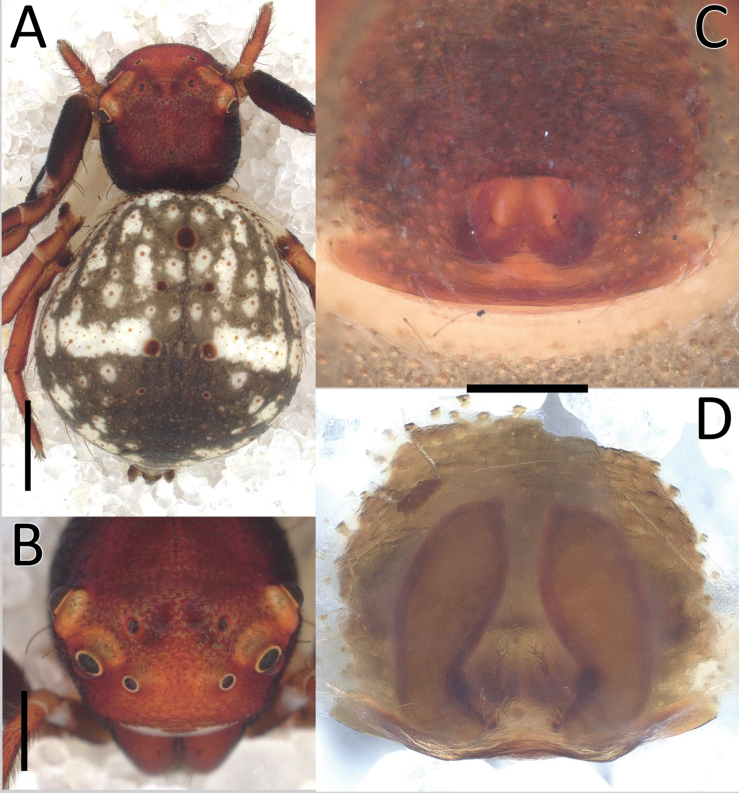
*Nyctimus kinabaluensis* Benjamin & Dhiya’ulhaq, sp. nov., from Sumatra. A. Female (2013_BF2.1_AraThom004N_001), habitus, dorsal view; B. Prosoma, frontal view; C. Epigynum, ventral view; D. Epigynum, dorsal view, cleared. Scale bars: 1 mm (A); 0.5 mm (B); 0.2 mm (C, D).

**Figure 7.**
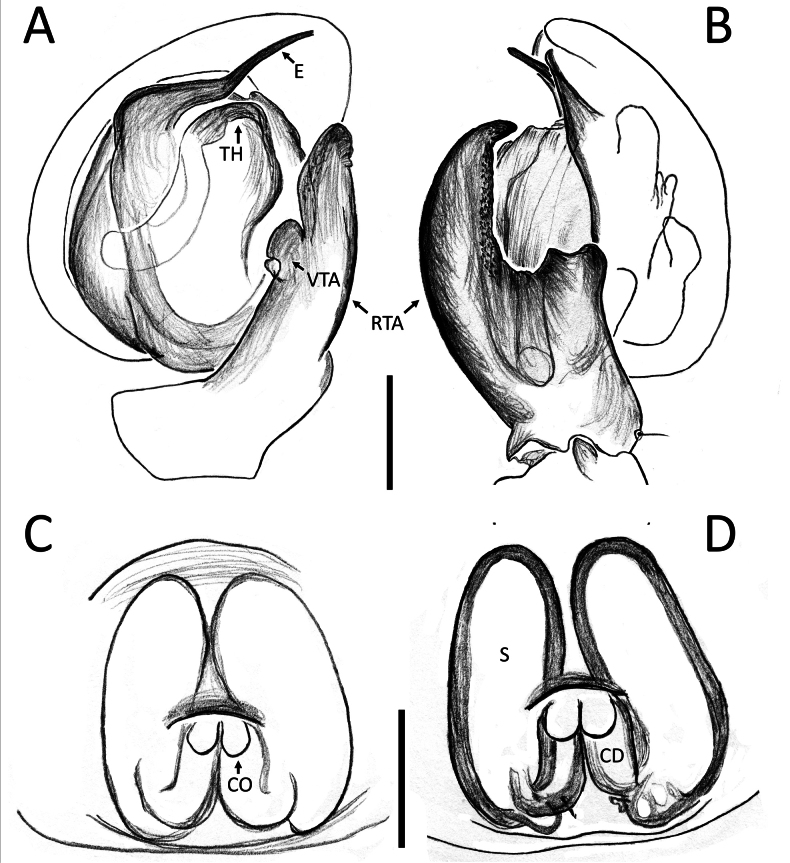
*Nyctimus kinabaluensis* Benjamin & Dhiya’ulhaq, sp. nov., from Borneo. A. Male (RMNH.ARA.17859), left palp, ventral view; B. ibid., retrolateral view; C. Female (RMNH.ARA.17862), epigynum, ventral view; D. ibid., dorsal view. Abbreviations: CD = copulatory duct; CO = copulatory opening; E = embolus; RTA = retrolateral tibial apophysis; S = spermatheca; TH = tegular hood; VTA = ventral tibial apophysis. Scale bars: 0.2 mm.

Palp (Fig. 7A, B): cymbium oval. Embolus elongated, tapering, with a 150° bend in the middle. Tegular hood small. RTA bilobed; ventral lobed very large, claw-shaped; dorsal lobe much smaller than ventral lobed, roughly triangular with an obtuse tip and a small triangular projection; VTA hook-shaped, small, attached medially on the RTA.

**Female** (RMNH.ARA.17859; Figs 5C, 7C, D). Total length: 3.38. Prosoma length 1.48; width 1.27. Opisthosoma length 1.90; width 1.70. Diameter of eyes: AME 0.07; ALE 0.17; PLE 0.14; PME 0.06. Interdistances between eyes: AME–AME 0.38; AME–ALE 0.12; ALE–ALE 0.74; PME–PME 0.39; PME–PLE 0.33; ALE–PLE 0.22; AME–PME 0.17; PLE–PLE 0.98. Leg measurements: leg I 4.6 (1.3, 0.4, 1.1, 1.0, 0.8). Habitus as in male.

Epigynum (Fig. 7C, D): exterior plate large, roughly square with rounded sides. Atrium oval. CO diagonally oriented, anteriorly facing. CD arched. Spermatheca long oval, with a slight bump anteriorly.

##### Distribution.

Brunei; Malaysia (Borneo: Sabah); Indonesia (Sumatra: Jambi) (Fig. 19).

#### ﻿ Nyctimus mutilloides

Taxon classificationAnimaliaAraneaeThomisidae

Benjamin & Dhiya’ulhaq sp. nov.

5A830190-ADB7-526B-8CE7-B63CF6B773FB

https://zoobank.org/04BD7F24-E0FF-49A5-A849-3C9430494FA4

Fig. 8

##### Etymology.

The specific epithet is derived from the latin name of the velvet ant family Mutillidae, and the Greek-derived suffix -*oides*, meaning “resembling” or “like.” Thus, *mutilloides* translates to “mutillid-like,” referring to the spider’s striking abdominal patterns resembling abdominal markings of certain trogaspidiine mutillids.

##### Type material.

***Holotype*.** Malaysia – Sabah State • ♂; Kinabalu National Park, Poring Hot Springs; 05°59'N, 116°42’E; elev. 600–700 m; 9 Feb. 1997; A. Floren leg.; canopy fogging; RMNHRMNH.ARA.17882.

##### Diagnosis.

**Figure 8.**
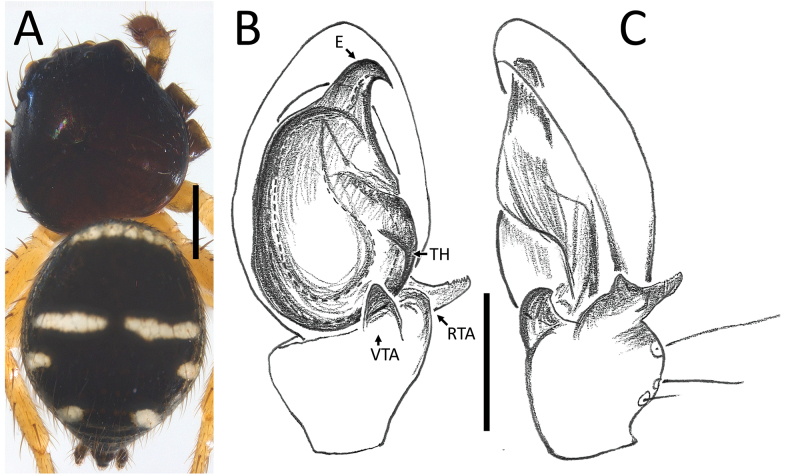
*Nyctimus mutilloides* Benjamin & Dhiya’ulhaq, sp. nov., male holotype (RMNH.ARA.17882). A. Habitus, dorsal view; B. Left palp ventral view; C. Left palp retrolateral view. Abbreviations: E = embolus; RTA = retrolateral tibial apophysis; TH = tegular hood; VTA = ventral tibial apophysis. Scale bars: 0.5 mm (A); 0.2 mm (B, C).

*Nyctimus mutilloides*, sp. nov., can be readily distinguished from its congeners by its vivid white markings on the opisthosoma, forming a pattern highly suggestive of mutillid wasps (spots on all congeners). Furthermore, the male palp has a claw-shaped embolus that juts out from the distal margin of the bulb (different in other congeners; Fig. 8B, C).

##### Description.

**Male** (holotype RMNH.ARA.17882; Fig. 8). Total length 2.80. Prosoma length 1.22; width 1.23. Opisthosoma length 1.57; width 1.22. Diameter of eyes: AME 0.07; ALE 0.12; PLE 0.10; PME 0.08. Interdistances between eyes: AME–AME 0.16; AME–ALE 0.11; ALE–ALE 0.39; PME–PME 0.2; PME–PLE 0.18; ALE–PLE 0.10; AME–PME 0.07; PLE–PLE 0.69. Prosoma cuboid, almost as long as wide, slightly flattened, weakly granulated, dark brown with darker margins, laterally adorned with long macrosetae; cephalic region elevated. Legs slender, pale yellow with indistinct annulations. No conspicuous spines observed. Opisthosoma oval, dorsoventrally flattened, with white spots and bands on black background.

Palp (Fig. 8B, C): cymbium slender, oval; tip rounded. Embolus claw-shaped, curved with a pointed tip, emerging distally from the tegulum. Tegulum oval; tegular hood wide, located retrolaterally near the base. RTA bilobed; both lobes roughly triangular; dorsal lobe with a serrated edge. VTA short, thumb-shaped, and slightly curved.

**Female.** Unknown.

##### Distribution.

Known only from the type locality (Malaysia: Borneo: Sabah) (Fig. 19).

##### Remarks.

The white dots and stripes on the opisthosoma of *N. mutilloides* are reminiscent of those of mutillid wasps, especially those of the tribe Trogaspidiini (see figs 26–37 in Okayasu 2023). Whether this constitutes a form of Batesian mimicry is unclear, but it is possible given that certain Gnaphosidae, Salticidae, and Clubionidae seem to mimic velvet ants (Nentwig 2008). Its presence in canopy fogging samples indicates an arboreal lifestyle, a noteworthy ecological trait within the genus. Further surveys and molecular data could shed light on its placement within *Nyctimus* and potential mimicry evolution in this group.

#### ﻿ Nyctimus quadripunctatus

Taxon classificationAnimaliaAraneaeThomisidae

Dhiya’ulhaq & Benjamin sp. nov.

14EA4F9E-30F1-5BC0-907E-E5D2595607ED

https://zoobank.org/EA408798-5CD6-4401-B44E-F6127489C4C1

Figs 9, 10, 11, 12

##### Etymology.

The specific epithet is taken from Latin, referring to the four white spots on the opisthosoma.

##### Type material.

***Holotype*.** Indonesia – Sumatra, Jambi Province • ♂; Batang Hari, Hutan Harapan Conservation Area; 02°11'15.3"S, 103°20'36.0"E; elev. 69 m; 7 Aug. 2013; J. Drescher leg.; canopy fogging in rainforest; GOET 2013_HF4.2_AraThom001N_001 (to be transferred to MZB). ***Paratypes*.** Indonesia – Sumatra, Jambi Province • 1♂1♀; Batang Hari, Hutan Harapan Conservation Area; 02°10'42.4"S, 103°19'58.2"E; elev. 54 m; 21 Jul. 2013; J. Drescher leg.; canopy fogging in rainforest; GOET 2013_HF3.2_AraThom001N_001–2 (to be transferred to MZB) • Malaysia – Sabah State • 1♂; Kinabalu National Park, Poring Hot Springs; 05°59'N, 116°42'E; elev. 600–700 m; 28 Mar. 1998; A. Floren leg.; canopy fogging on *Barringtonia* tree, in primary forest; RMNHRMNH.ARA.17858. • 1♂; Kota Marudu, Kampong Sorinsim; 06°06'N, 116°50'E; elev. 600–700 m; 2 Mar. 1997; A. Floren leg.; canopy fogging in 15 years old secondary forest; RMNHRMNH.ARA.17860.

##### Other material examined.

Malaysia – Sabah State • 1♂; Kinabalu National Park, Poring Hot Springs; 05°59'N, 116°42'E; elev. 600–700 m; 27 Feb. 1996; A. Floren leg.; canopy fogging on *Aporosa subcaudata* tree, in primary forest; RMNHRMNH.ARA.17857.

##### Diagnosis.

Males are easily distinguished from other congeners by the anvil-shaped RTA ending in two acute lobes (Fig. 11A, B). Females can be distinguished from *N. bistriatus* by the much thinner, shorter CD and paired CO (Figs 10C, D, 11C, D; vs single CO in *N. bistriatus*, Figs 2C, 3C), and from *N. rendang*, sp. nov., by the spermatheca lacking a prominent posterior extension (Figs 10D, 11D; vs Figs 14D, 15D).

##### Description.

**Figure 9.**
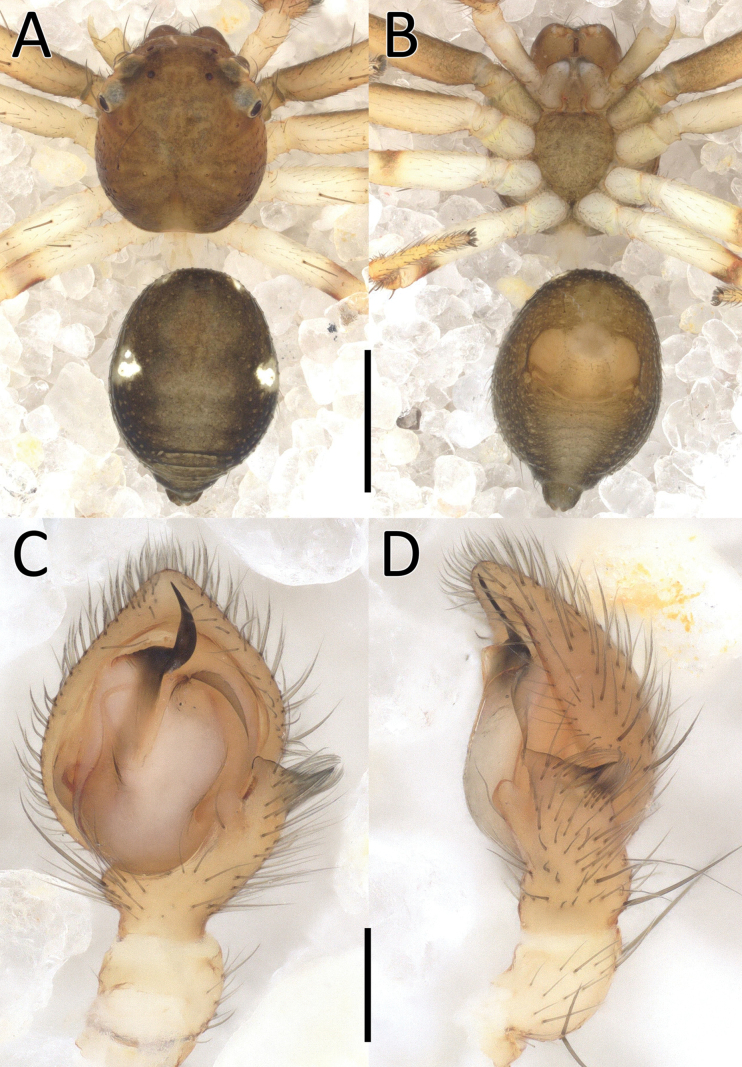
*Nyctimus quadripunctatus* Dhiya’ulhaq & Benjamin, sp. nov., male holotype (AraThom001N_2013_HF4.2_001). A. Habitus, dorsal view; B. Habitus ventral view; C. Right palp, mirrored, ventral view; D. Right palp, mirrored, retrolateral view. Scale bars: 1 mm (A, B); 0.2 mm (C, D).

**Male** (holotype 2013_HF4.2_AraThom001N_001; Figs 9, 11A, B). Total length 2.95. Prosoma length 1.35; width 1.23. Opisthosoma length 1.60; width 1.22. Diameter of eyes: AME 0.07; ALE 0.16; PLE 0.16; PME 0.06. Inter­distances between eyes: AME–AME 0.38; AME–ALE 0.08; ALE–ALE 0.68; PME–PME 0.34; PME–PLE 0.32; ALE–PLE 0.19; AME–PME 0.18; PLE–PLE 0.98. Clypeus height 0.00. Leg measurements: leg I 4.25 (1.19, 0.49, 1.06, 0.90, 0.61); leg II 3.89 (1.11, 0.42, 0.90, 0.81, 0.65); leg III 2.63 (0.86, 0.35, 0.57, 0.49, 0.36); leg IV 2.74 (0.83, 0.27, 0.66, 0.52, 0.46). Prosoma cuboid, almost as long as wide, box-shaped, weakly granulated, highest at mid-point, light brown; lateral margins furnished with a row of long macrosetae. Lateral eyes large, situated on tubercles on the lateral edge of the prosoma. Chelicerae, endites, and labium light brown. Sternum light brown. Legs I light brown; others yellow-brown, with sparse spines and black setae. Opisthosoma oval, brown, with two pairs of large white spots. Spinnerets brown.

**Figure 10.**
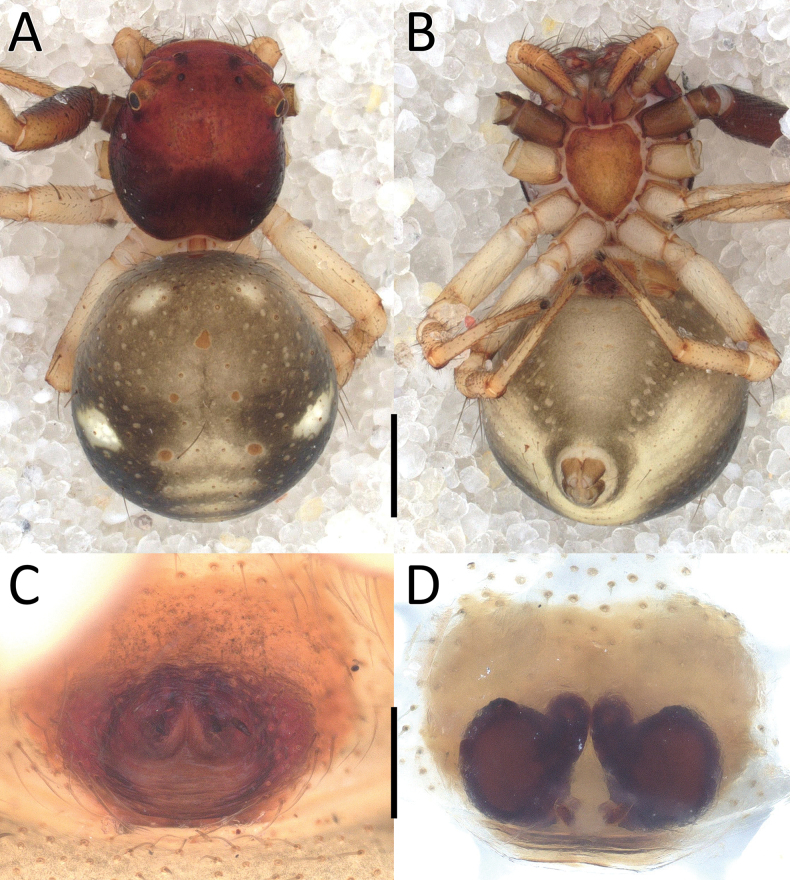
*Nyctimus quadripunctatus* Dhiya’ulhaq & Benjamin, sp. nov., female paratype (AraThom001N_2013_HF3.2_001). A. Habitus, ventral view; B. Habitus, dorsal view; C. Epigynum, ventral view; D. Epigynum, dorsal view, cleared. Scale bars: 1 mm (A, B); 0.2 mm (C, D).

**Figure 11.**
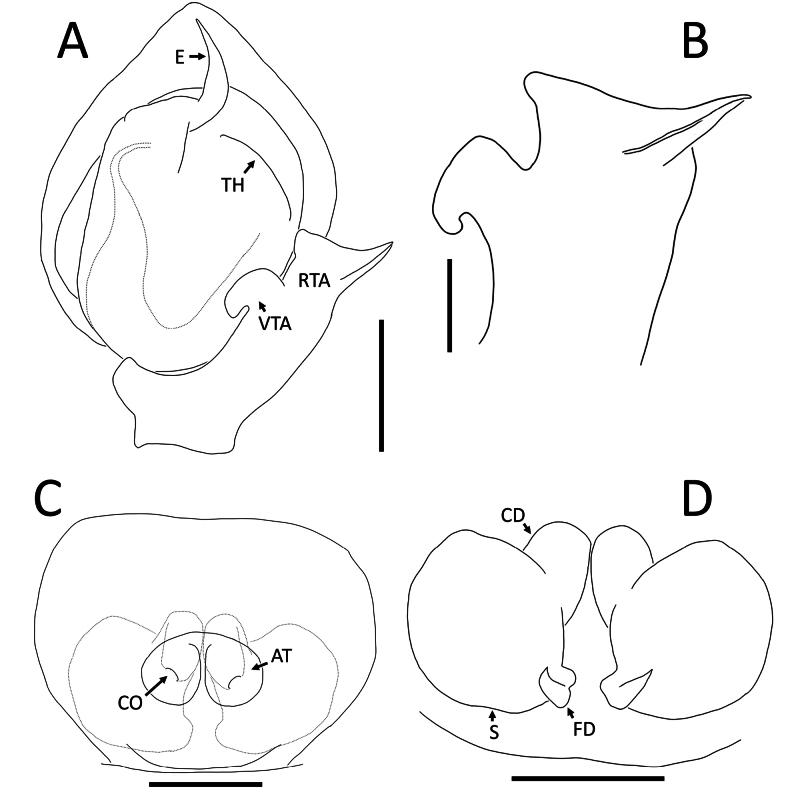
*Nyctimus quadripunctatus* Dhiya’ulhaq & Benjamin, sp. nov. A. Male holotype (AraThom001N_2013_HF3.2_001), right palp, mirrored, ventral view; B. ibid., RTA, ventro-retrolateral view; C. Female paratype (AraThom001N_2013_HF3.2_001), epigynum, ventral view; D. ibid., dorsal view. Abbreviations: CD = copulatory duct; CO = copulatory opening; E = embolus; RTA = retrolateral tibial apophysis; S = spermatheca; TH = tegular hood; VH = ventral hook. Scale bars: 0.2 mm.

Palp (Figs 9C, D, 11A, B): cymbium oval. Embolus sickle-shaped; base slightly wrinkled. Tegular hood very wide. RTA large, rectangular, anvil-shaped; dorsally bifurcated, ending into two long, acute lobes, hidden behind a brush of setae; VTA hook-shaped attached medially on RTA.

**Female** (paratype 2013_HF3.2_AraThom001N_002; Figs 10, 11C, D). Total length 4.81. Prosoma length 1.95; width 1.77. Opisthosoma length 2.86; width 2.65. Diameter of eyes: AME 0.09; ALE 0.20; PLE 0.18; PME 0.07. Interdis­tances between eyes: AME–AME 0.53; AME–ALE 0.14; ALE–ALE 0.97; PME–PME 0.52; PME–PLE 0.39; ALE–PLE 0.23; AME–PME 0.24; PLE–PLE 1.37. Leg measurements: leg I 5.47 (1.66, 0.57, 1.34, 1.13, 0.77); leg II -; leg III 3.69 (1.22, 0.47, 0.87, 0.63, 0.50); leg IV 4.12 (1.36, 0.46, 0.96, 0.76, 0.58). Habitus as in males, except prosoma and leg I reddish brown.

Epigynum (Figs 10C, D, 11C, D): exterior plate large, hexagonal-shaped with wide anterior side. Atrium conspicuous, inverted heart-shaped with a separation in the middle. CO diagonally oriented, posteriorly facing. CD arch-shaped. Spermatheca elongated, peanut-shaped, with a small bump anteriorly.

##### Distribution.

Malaysia (Borneo: Sabah); Indonesia (Sumatra: Jambi) (Fig. 19).

##### Remarks.

**Figure 12.**
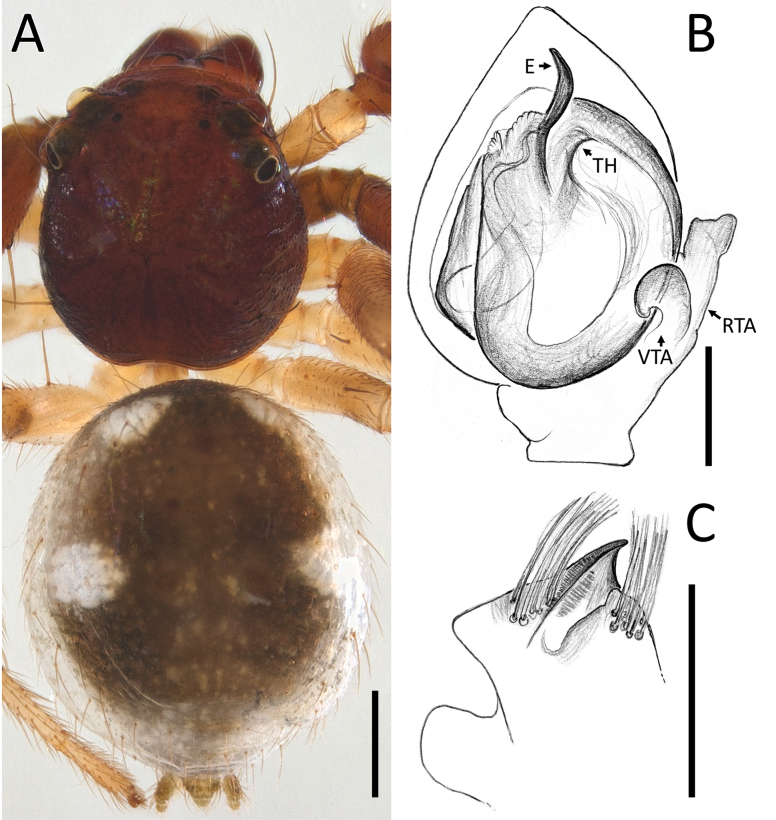
*Nyctimus quadripunctatus* Dhiya’ulhaq & Benjamin, sp. nov., non-type male specimen from Borneo (RMNH.ARA.17857). A. Habitus, dorsal view; B. Left palp, ventral view; C. Left palpal tibia, retrolateral view. Abbreviations: E = embolus; RTA = retrolateral tibial apophysis; TH = tegular hood; VH = ventral hook. Scale bars: 0.5 mm (A); 0.2 mm (B, C).

Specimens from Borneo show slight differences in shape of embolus and dorsal tip of RTA (Fig. 12B, C).

#### ﻿ Nyctimus rendang

Taxon classificationAnimaliaAraneaeThomisidae

Dhiya’ulhaq & Benjamin sp. nov.

043239DA-7E40-5B97-9B41-29D6254CD462

https://zoobank.org/D674C659-6FE3-41A2-9484-300E4C2A918D

Figs 13, 14, 15

##### Etymology.

The specific epithet refers to a meat dish associated with the cuisine of the Minangkabau people from West Sumatra. The meat is slow-cooked in a plethora of spices, giving it a dark colouration. The dark colour of *N. rendang*, sp. nov., is reminiscent of the colour of this dish.

##### Type material.

***Holotype*.** Indonesia – Sumatra, Jambi Province • ♂; Sarolangun, Air Hitam, Desa Baru; 02°01'49.5"S, 102°46'14.8"E; elev. 57 m; 12 Jul. 2013; J. Drescher leg.; canopy fogging in jungle rubber plantation; GOET 2013_BJ6.1_AraThom010N_001 (to be transferred to MZB). ***Paratypes*.** Indonesia – Sumatra, Jambi Province • 1♂1♀; same data as holotype; GOET 2013_BJ6.1_AraThom010N_002, 003 (to be transferred to MZB). • 2♂♂; Batang Hari, Bajubang, Singkawang; 01°47'07.9"S, 103°16'37.4"E; elev. 56 m; 18 Jun. 2013; J. Drescher leg.; canopy fogging in jungle rubber plantation; GOET 2013_HJ4.2_AraThom010N_001, 002 (to be transferred to ZMH).

##### Other material examined.

Indonesia – Sumatra, Jambi Province • 1♀; Batang Hari, Hutan Harapan Conservation Area; 02°11'15.3"S, 103°20'36.0"E; elev. 69 m; 7 Aug. 2013; J. Drescher leg.; canopy fogging in rainforest; GOET 2013_HF4.1_AraThom036N_001.

##### Diagnosis.

Males are similar to *N. quadripunctatus*, sp. nov., in having a sickle-shaped embolus and in the general shape of the bulb, but the VTA is digitiform and free (Fig. 15A, B; vs hook-shaped, attached to RTA in *N. quadripunctatus*, Fig. 11A, B) and the RTA possesses a pillar-shaped ventral lobe and paired, thick spines (vs both absent). Females are distinct in having the spermathecae with a posterior extension and a short, barely noticeable CD (Fig. 11D).

##### Description.

**Figure 13.**
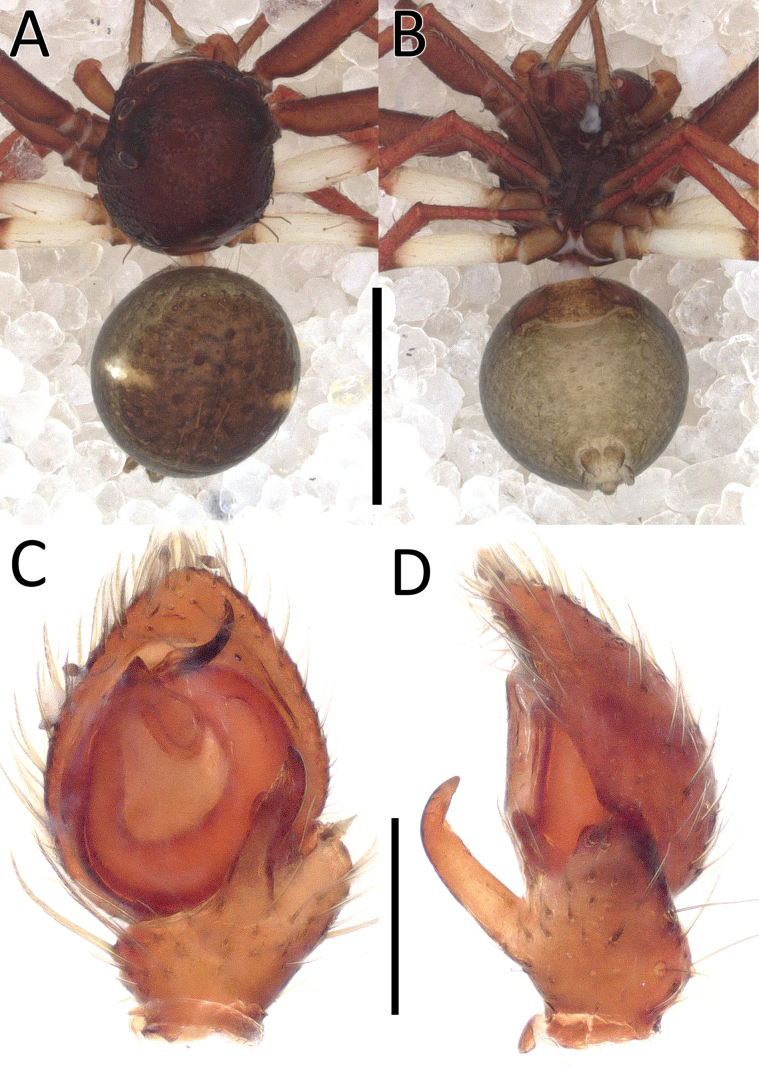
*Nyctimus rendang* Dhiya’ulhaq & Benjamin, sp. nov., male holotype (AraThom010N_2013_BJ6.1_001). A. Habitus, dorsal view; B. Habitus, ventral view; C. Left palp, ventral view; D. Left palp, retrolateral view. Scale bars: 1 mm (A, B); 0.2 mm (C, D).

**Male** (holotype 2013_BJ6.1_AraThom010N_001; Figs 13, 15A, B). Total length 2.41. Prosoma length 1.16; width 1.03. Opisthosoma length 1.25; width 1.21. Diameter of eyes: AME 0.07; ALE 0.15; PLE 0.14; PME 0.05. Interdistances between eyes: AME–AME 0.35; AME–ALE 0.08; ALE–ALE 0.62; PME–PME 0.32; PME–PLE 0.24; ALE–PLE 0.17; AME–PME 0.19; PLE–PLE 0.77. Leg measurements: leg I 3.36 (0.90, 0.29, 0.92, 0.75, 0.50); leg II 3.46 (1.04, 0.32, 0.84, 0.75, 0.51); leg III 2.11 (0.67, 0.20, 0.54, 0.38, 0.32); leg IV 2.25 (0.75, 0.18, 0.59, 0.42, 0.31). Prosoma cuboid, almost as long as wide, dark reddish brown; surface weakly granulated; laterally adorned with long macrosetae, each arising from a small tubercle. Chelicerae, and sternum coloured as pro­soma. Endites brown with white tip. Front legs brown; back legs brown except for the white femora. Opisthosoma round, dark brown with paired white bars in the middle; dorsally covered with scutum; five large sigillae present, arranged in a triangular formation.

Palp (Figs 13C, D, 15A, B): cymbium oval. Embolus sickle-shaped. Tegulum with a small triangular projection next to the embolus; tegular hood small. RTA bilobed; ventral lobe finger-shaped; dorsal lobe wide trapezoid, apically with a pair of short, thick spines. VTA long, finger-shaped, apically flexed.

**Figure 14.**
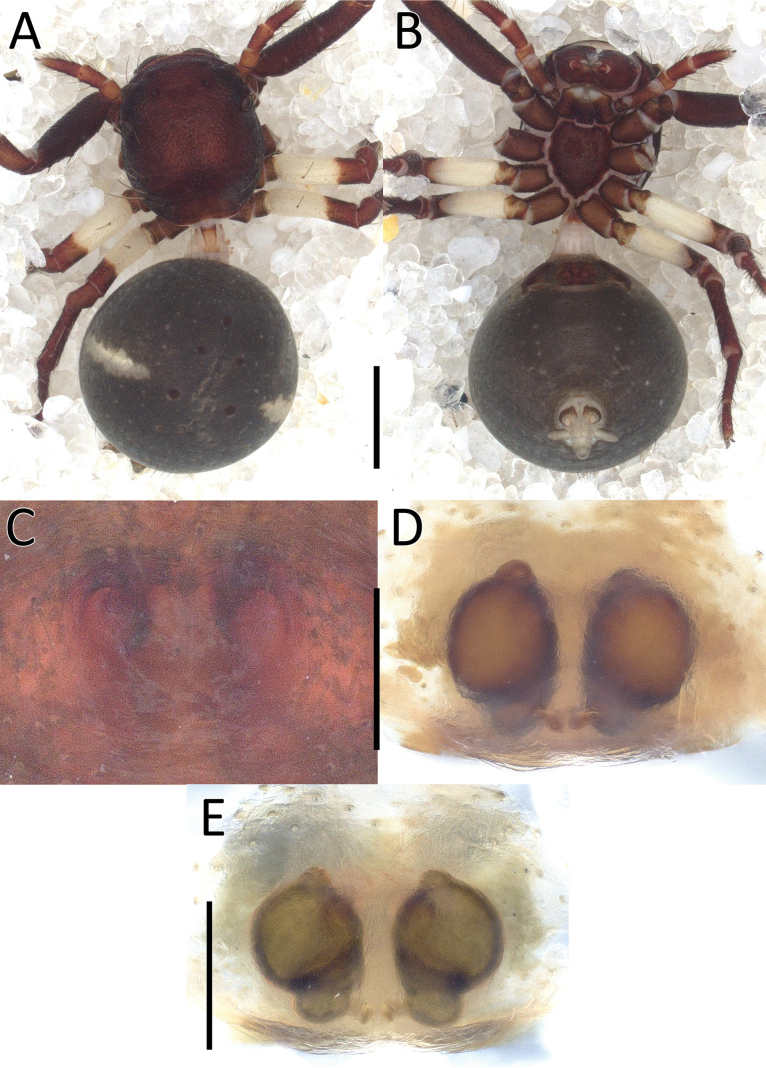
*Nyctimus rendang* Dhiya’ulhaq & Benjamin, sp. nov., female paratype (AraThom010N 2013 BJ6.1 003). A. Habitus, dorsal view; B. Habitus, ventral view; C. Epigynum, ventral view; D. Epigynum, dorsal view; E. Epigynum, dorsal view, cleared. Scale bars: 1 mm (A, B); 0.2 mm (C–E).

**Figure 15.**
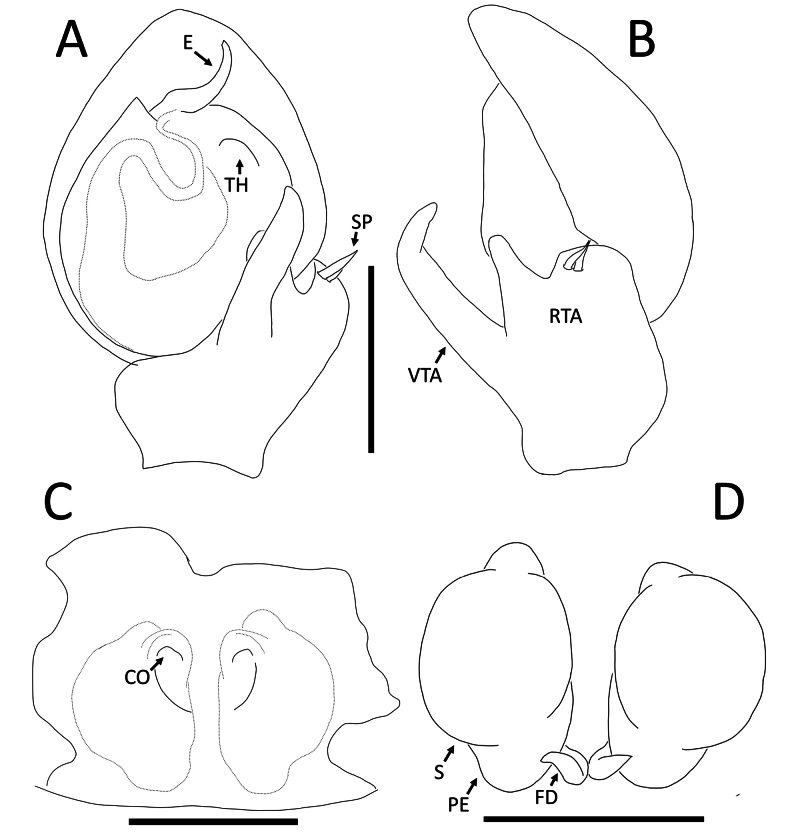
*Nyctimus rendang* Dhiya’ulhaq & Benjamin, sp. nov. A. Male holotype (AraThom010N 2013 BJ6.1 001), left palp, ventral view; B. ibid., left palp, retrolateral view; C. Female paratype (AraThom010N_2013_BJ6.1_003), epigynum, dorsal view; D. ibid., epigynum, ventral view. Abbreviations: CO = copulatory opening; E = embolus; PE = posterior extension of spermatheca; RTA = retrolateral tibial apophysis; SP = spine; S = spermatheca; TH = tegular hood; VTA = ventral tibial apophysis. Scale bars: 0.2 mm.

**Female** (paratype AraThom010N_2013_BJ6.1_003; Figs 14, 15C, D). Total length 3.86. Prosoma length 1.67; width 1.43. Opisthosoma length 2.19; width 2.21. Diameter of eyes: AME 0.08; ALE 0.18; PLE 0.19; PME 0.07. Interdis­tances between eyes: AME–AME 0.55; AME–ALE 0.12; ALE–ALE 0.90; PME–PME 0.45; PME–PLE 0.34; ALE–PLE 0.23; AME–PME 0.23; PLE–PLE 1.11. Leg measurements: leg I 4.85 (0.71, 1.01, 1.18, 0.49, 1.46); leg II -; leg III 3.05 (1.00, 0.30, 0.75, 0.54, 0.46); leg IV 3.41 (1.14, 0.32, 0.83, 0.65, 0.47). Habitus as in male, except opisthosoma not covered in scutum.

Epigynum (Fig. 14C, D, 15C, D): exterior plate with irregular border. Atrium inconspicuous. CO semicircular, posteriorly facing. CD very short. Spermatheca oval, anteriorly with a large bump, posteriorly extended.

##### Distribution.

Indonesia (Sumatra: Jambi) (Fig. 19).

##### Remarks.

One specimen shows rounded rather than oval spermathecae (Fig. 14E) and is, thus, tentatively placed here, as no males from the same locality were found.

#### ﻿ Nyctimus saksang

Taxon classificationAnimaliaAraneaeThomisidae

Dhiya’ulhaq & Benjamin sp. nov.

CD696F87-6463-5550-B847-8967D3B46BE2

https://zoobank.org/D731CC5A-13CF-480B-9894-01BCC9A42F9E

Figs 16, 17

##### Etymology.

The specific epithet refers to a meat-stew dish associated with the Batak people from North Sumatra. The meat is cooked in blood, giving it a dark colouration. The dark colour of *Nyctimus saksang* Benjamin & Dhiya’ulhaq, sp. nov., is reminiscent of the colour of this dish.

##### Type material.

***Holotype*.** Indonesia – Sumatra, Jambi Province • ♂; Batang Hari, Hutan Harapan Conservation Area; 02°09'48.9"S, 103°20'04.4"E; elev. 73 m; 6 Jul. 2013; J. Drescher leg.; canopy fogging in rainforest; GOET 2013_HF2.1_AraThom036N_001 (to be transferred to MZB).

##### Diagnosis.

**Figure 16.**
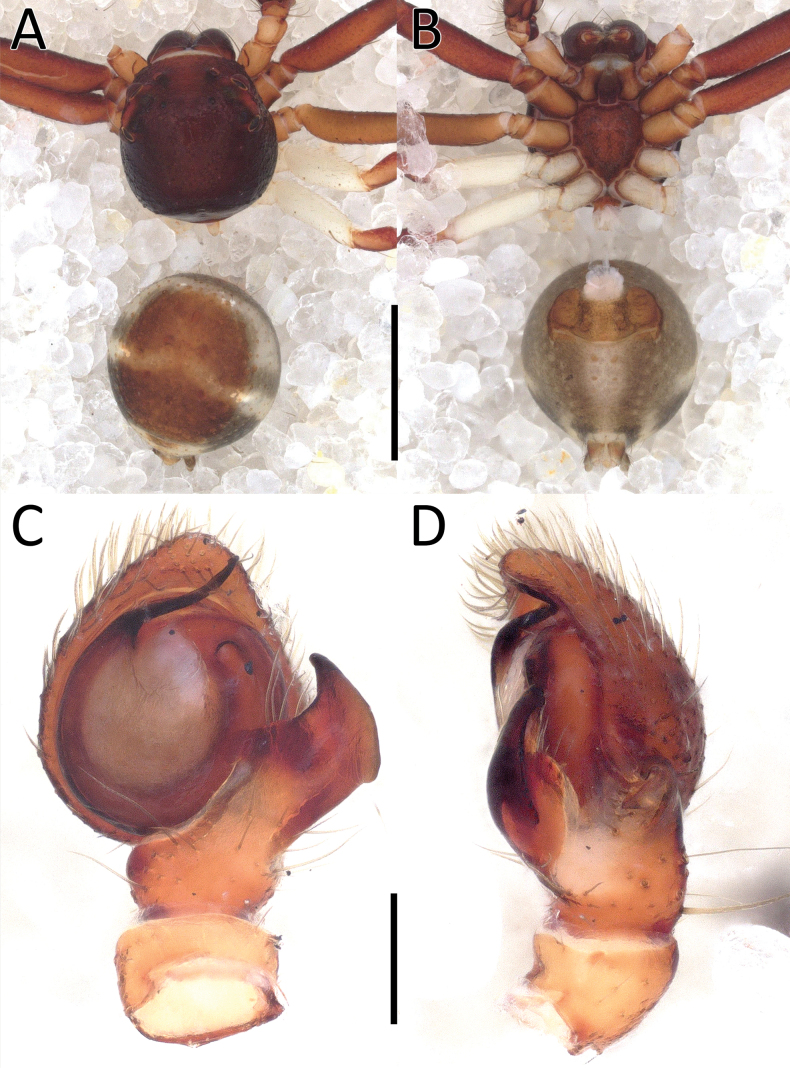
*Nyctimus saksang* Dhiya’ulhaq & Benjamin, sp. nov. male holotype (AraThom036N 2013 HF2.1 001). A. Habitus, dorsal view; B. Habitus, ventral view; C. Left palp, ventral view; D. Left palp, retrolateral view. Scale bars: 1 mm (A, B); 0.2 mm (C, D).

**Figure 17.**
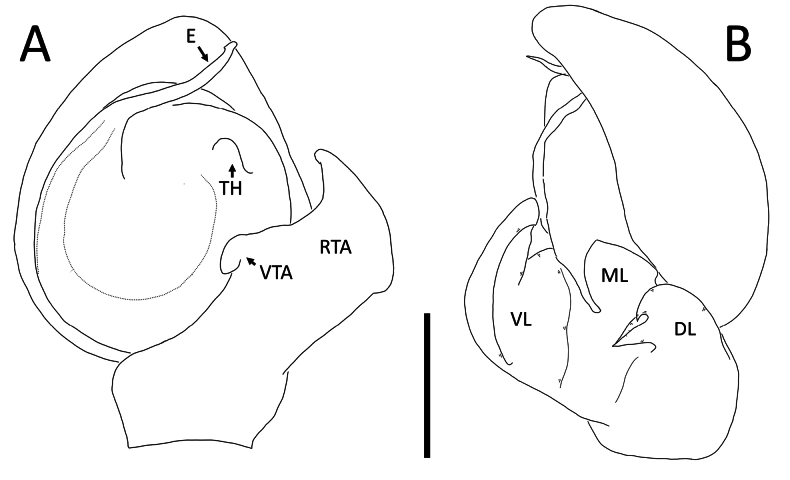
*Nyctimus saksang* Dhiya’ulhaq & Benjamin, sp. nov. male holotype (AraThom036N 2013 HF2.1 001). A. Left palp, ventral view; B. Left palp, retrolateral view. Abbreviations: DL = dorsal lobe; E = embolus; ML = medial lobe; RTA = retrolateral tibial apophysis; TH = tegular hood; VTA = ventral tibial apophysis; VL = ventral lobe. Scale bar: 0.2 mm.

Males can be easily distinguished from all other congeners by the very wide, axe-shaped RTA which consists of three large lobes (Figs 16C, D, 17).

##### Description.

**Male** (holotype 2013_HF2.1_AraThom036N_001; Figs 16, 17). Total length 3.11. Prosoma length 1.50; width 1.25. Opisthosoma length 1.61; width 1.40. Diameter of eyes: AME 0.08; ALE 0.17; PLE 0.14; PME 0.05. Inter­distances between eyes: AME–AME 0.40; AME–ALE 0.10; ALE–ALE 0.73; PME–PME 0.38; PME–PLE 0.33; ALE–PLE 0.21; AME–PME 0.22; PLE–PLE 0.95. Leg measurements: leg I 4.20 (1.20, 0.44, 1.05, 0.89, 0.62); leg II 3.74 (1.10, 0.34, 0.91, 0.78, 0.61); leg III 2.78 (0.83, 0.37, 0.67, 0.52, 0.39); leg IV 2.87 (0.88, 0.26, 0.70, 0.60, 0.43). Prosoma cuboid, almost as long as wide, dark reddish brown; surface weakly granulated; laterally adorned with long macrosetae, each arising from a small tubercle. Chelicerae and sternum coloured as prosoma. Endites brown with white tip. Front legs brown; back legs brown except for the white femora. Opisthosoma round, dark brown with paired white bars in the middle that are almost touching each other; anteriorly with a white border; posteriorly with a white patch; dorsally covered with scutum; five large sigillae present, arranged in a triangular formation.

Palp (Figs 16C, D, 17): cymbium oval. Embolus elongated, tapering; tip with a slight flange. Tegular hood small. RTA trilobed; ventral lobed finger-shaped; median lobe triangular, with dorsal lobe axe-shaped; dorsal edge slightly folded. VTA hook-shaped, small, attached medially on the ventral lobe of RTA.

**Female.** Unknown.

##### Distribution.

Only known from the type locality (Indonesia: Sumatra: Jambi) (Fig. 19).

#### ﻿ Zametopina

Taxon classificationAnimaliaAraneaeThomisidae

Simon, 1909

68082F37-4A2F-5D83-A03E-FE75127061E0

##### Type species.

*Zametopina calceata* Simon, 1909.

##### Diagnosis.

**Figure 18.**
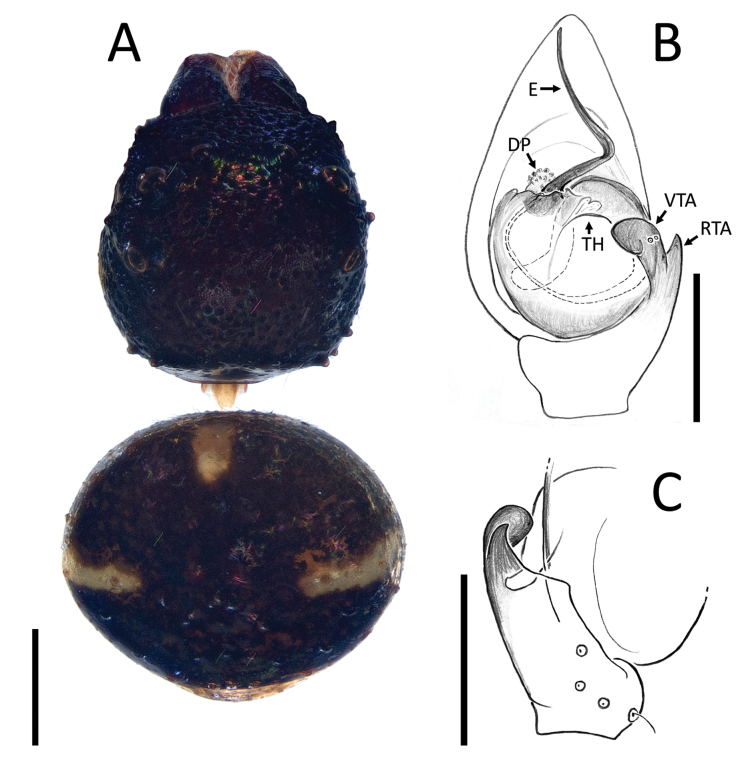
*Zametopina wanliae* Lin & Li, 2023, male (MCZ6652). A. Habitus, dorsal view; B. Left palp, ventral view; C. Left palpal tibia, retrolateral view. Abbreviations: DP = denticulate process; E = embolus; RTA = retrolateral tibial apophysis; TH = tegular hood; VTA = ventral tibial apophysis. Scale bars: 0.5 mm (A); 0.2 mm (B, C).

*Zametopina* is similar to *Nyctimus* in habitus, differing in the presence of a unique denticulate process next to the embolus on the male palp (DP in Fig. 18B). No other oriental thomisid genus possesses this structure. For a detailed diagnosis see Tang et al. (2010).

##### Species composition.

*Zametopina calceata* Simon, 1909 and *Zametopina wanliae* Lin & Li, 2023.

#### ﻿ Zametopina calceata

Taxon classificationAnimaliaAraneaeThomisidae

Simon, 1909

6487E2EB-928A-57BE-84E8-F71297687052

Zametopina calceata Simon, 1909: Simon 1909: 123; Tang et al. (2010): 66, figs 1−18.

##### Type material.

***Holotype*.** Vietnam – Cao Bằng Province • ♂; “Boa Luc, dans Haut-Tonkin, à l’Ouest de Cao-Bang et prés la frontière du Yun-Nan et du Kuang-Si” (probably Bảo Lạc District); 1906 or 1907; de Pelacot leg.; MNHN 22937. Examined.

##### Diagnosis.

See Lin et al. (2023).

##### Description.

See Tang et al. (2010).

##### Distribution.

**Figure 19.**
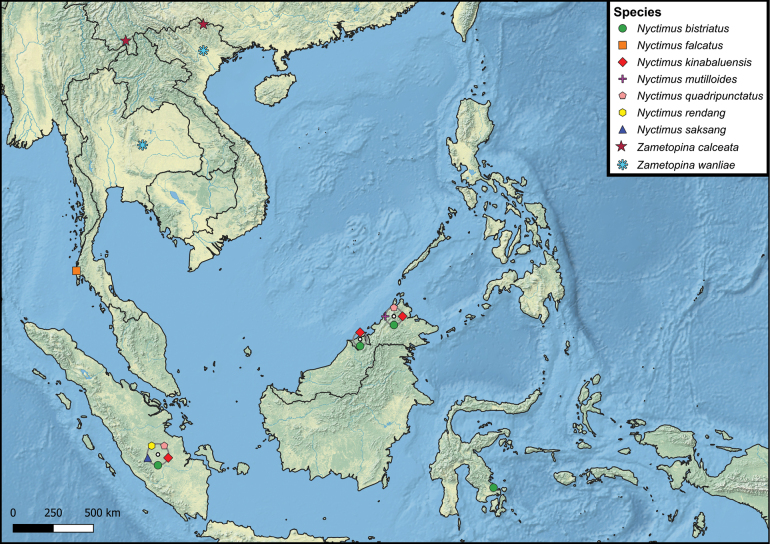
Distribution of all described *Nyctimus* and *Zametopina* (Araneae: Thomisidae) species. Grey circles surrounding white dots indicate species collected from the same or very closely located sampling points in Brunei, Sabah (Malaysia), and Jambi (Indonesia).

China (Yunnan), Vietnam (Cao Bằng) (Fig. 19).

#### ﻿ Zametopina wanliae

Taxon classificationAnimaliaAraneaeThomisidae

Lin & Li, 2023

83804B74-7CD8-5BBE-9E96-196B32D20079

Fig. 18

Zametopina wanliae Lin & Li, 2023 in Lin et al. (2023): 79, figs 68A, B, 69I.

##### Material examined.

Thailand – Chaiyaphum Province • 2♂♂; Tat Ton National Park, Tat Fa Waterfall; 15°56.458'N, 102°5.855'E; elev. 600–700 m; 7. And 8. Mar. 2007; Tawit Jarupan et al. leg.; canopy fogging in rainforest; MCZ MCZ128814, MCZ6652.

##### Diagnosis.

See Lin et al. (2023).

##### Description.

**Male** (MCZ6652; Fig. 18). Total length 2.44. Prosoma length 1.22; width 1.14. Opisthosoma length 1.22; width 1.40. Diameter of eyes: AME 0.09; ALE 0.15; PLE 0.12; PME 0.09. Interdistances between eyes: AME–AME 0.30; AME–ALE 0.13; ALE–ALE 0.69; PME–PME 0.37; PME–PLE 0.35; ALE–PLE 0.23; AME–PME 0.07; PLE–PLE 0.85.

For a complete description, see Lin et al. (2023).

##### Distribution.

Vietnam (Vĩnh Phúc), Thailand (Chaiyaphum [new record]) (Fig. 19).

## ﻿Discussion

A key question concerns whether the females assigned to *Nyctimus kinabaluensis*, sp. nov., are conspecific with the male holotype. These are the only female specimens of *Nyctimus* included in this study and possess an oval spermatheca and a unique configuration of the copulatory ducts. Interestingly, similar features are observed in the female of *Zametopina calceata*, as illustrated by Tang et al. (2010), raising the possibility of a male–female mismatch. Nevertheless, we consider such a mix-up unlikely given the strong resemblance in habitus between the males and females. The overall somatic morphology is consistent, and although the genitalia bear superficial similarities to *Z. calceata*, we regard the differences as sufficient to support the recognition of a distinct species.

This observation leads to a broader taxonomic consideration: whether all species currently attributed to *Nyctimus* should indeed belong to the same genus. Most species share a remarkably uniform somatic appearance, dark colouration with white markings, a cuboid, granulated prosoma furnished with long setae, and, in males, a prominent scutum. However, genital morphology across the group is highly variable. The type species, *N. bistriatus*, is morphologically divergent in several key respects: it has widely spaced median eyes, an exceptionally long embolus, a raspy retrolateral tibial apophysis (RTA), and a broad, elongate copulatory duct with a single copulatory opening. These traits set it apart from the other species and raise doubts about the coherence of the genus as currently defined.

All *Nyctimus* species described here exhibit a weakly granulated, dark-brown prosoma that peaks in height at mid-length and possesses lateral margins lined with long macrosetae, an unusual feature among Oriental Thomisidae. Comparable setae are known only from *Tagulis mystacinus* Simon, 1895, a species endemic to Sri Lanka, whose closest relatives are found in Africa (Benjamin and Ranasinghe 2019).

All species discussed here possess a highly modified ventral tibial apophysis (VTA) that varies in shape and size. Most known thomisids, informally referred to as “higher thomisids” or the *Thomisus* clade (Benjamin 2011; Ramírez 2014), represent the bulk of the family’s species diversity. These higher thomisids exhibit a distinct male palpal morphology, characterised by a disk-shaped tegulum (character 11 in Benjamin 2011) and a hook-shaped VTA, which appears to function as a guiding mechanism during hematodochal expansion and rotation (Huber 1994; character 320 in Ramírez 2014).

However, this specific form of VTA is only observed in *Nyctimus bistriatus*, *N. quadripunctatus* sp. nov., and *Zametopina wanliae*. In most of the species discussed here, the VTA is modified in its size, shape, and position on the apical surface of the tibia. In the absence of evidence to the contrary, the most parsimonious assumption is that all these apical ventral apophyses are homologous. If this character system were to be coded for a future phylogenetic matrix, we would propose the following coding scheme: Character #n: VTA, 0, present; 1, absent. Character #n+1: Position, distance from the tibial apex. Character #n+2: Relative size of the VTA.

Among the species discussed, *N. mutilloides*, sp. nov., is particularly distinctive. The male, for which no female is yet known, displays white dots and bands across the opisthosoma that strongly resemble the aposematic markings of mutillid wasps, whereas other congeners only bear spotted abdominal patterns. Additionally, the male palp is unique: the embolus is beak-shaped and projects prominently from the distal margin of the bulb. To our knowledge, no other thomisids in the Oriental region exhibit such a palp structure. Although this may indicate a distinct lineage within the genus, further material, particularly molecular data and female specimens, is required to test whether this species merits recognition at the genus level.

Clarifying these taxonomic relationships will require molecular phylogenetic data. At present, the evolutionary affinities and higher-level placement of these species remain uncertain. A robust phylogenetic framework would provide essential insights into lineage divergence, character evolution, and genus-level boundaries. In the absence of such data, hypotheses regarding species interrelationships remain speculative and lack firm genetic or evolutionary support. Future studies incorporating both morphological and molecular evidence will be crucial for resolving these questions and establishing a stable classification for this group of crab spiders.

## Supplementary Material

XML Treatment for Nyctimus

XML Treatment for Nyctimus bistriatus

XML Treatment for Nyctimus falcatus

XML Treatment for Nyctimus kinabaluensis

XML Treatment for Nyctimus mutilloides

XML Treatment for Nyctimus quadripunctatus

XML Treatment for Nyctimus rendang

XML Treatment for Nyctimus saksang

XML Treatment for Zametopina

XML Treatment for Zametopina calceata

XML Treatment for Zametopina wanliae

## ﻿Additional information

### Use of AI

No use of AI was reported.

### Author ORCIDs

Suresh P. Benjamin https://orcid.org/0000-0003-4666-0330

Naufal Urfi Dhiya’ulhaq https://orcid.org/0000-0001-6563-4643

Damayanti Buchori https://orcid.org/0000-0002-2843-0737

Purnama Hidayat https://orcid.org/0000-0001-9507-6275

Stefan Scheu https://orcid.org/0000-0003-4350-9520

Jochen Drescher https://orcid.org/0000-0002-5162-9779

### Data availability

All of the data that support the findings of this study are available in the main text.

## References

[B1] Benjamin SP (2011) Phylogenetics and comparative morphology of crab spiders (Araneae: Dionycha, Thomisidae). Zootaxa 3080(1): 1–108. 10.11646/zootaxa.3080.1.1

[B2] Benjamin SP Ranasinghe UGSL (2019) Redescription of *Tagulis granulosus* (Araneae: Thomisidae) from Sierra Leone. Arachnology 18(1): 22–23. 10.13156/arac.2018.18.1.22

[B3] Dhiya’ulhaq NU Ramos Gutierrez DE Nazarreta R Lia M Pakpahan B Buchori D Scheu S Drescher J (2024) Spiders of Jambi: a Guide to the EFForTS Collection. e-Publishing Penerbrit BRIN, Jakarta. 10.55981/brin.824

[B4] Drescher J Rembold K Allen K Beckschäfer P Buchori D Clough Y Faust H Fauzi AM Gunawan D Hertel D Irawan B Jaya INS Klarner B Kleinn C Knohl A Kotowska MM Krashevska V Krishna V Leuschner C Lorenz W Meijide A Melati D Nomura M Pérez-Cruzado C Qaim M Siregar IT Steinebach S Tjoa A Tscharntke T Wick B Wiegand K Kreft H Scheu S (2016) Ecological and socio-economic functions across tropical land use systems after rainforest conversion. Philosophical Transactions of the Royal Society of London. Series B, Biological Sciences 371(1694): 20150275. 10.1098/rstb.2015.0275 PMC4843696 27114577

[B5] Huber BA (1994) Genital bulb muscles in entelegyne spiders. The Journal of Arachnology 22: 75–76.

[B6] Ileperuma Arachchi IS Benjamin SP (2019) Twigs that are not twigs: Phylogenetic placement of crab spiders of the genus *Tmarus* of Sri Lanka with comments on the higher level phylogeny of Thomisidae. Invertebrate Systematics 33: 575–595.

[B7] Lehtinen PT (2016) Significance of oriental taxa in phylogeny of crab spiders (Thomisidae s.lat. and Stiphropodidae). Indian Journal of Arachnology 5: 143–171.

[B8] Lin YJ Li SQ Pham DS (2023) Taxonomic notes on some spider species (Arachnida: Araneae) from China and Vietnam. Zoological Systematics 48(1): 1–99. 10.11865/zs.2023101

[B9] Nentwig W (2008) A mimicry complex between mutillid wasps (Hymenoptera: Mutillidae) and spiders (Araneae). Studies on Neotropical Fauna and Environment 20(2): 113–116. 10.1080/01650528509360679

[B10] Okayasu J (2023) Love is in the air and beyond the ocean: taxonomic review of *Neotrogaspidia* Lelej (Hymenoptera: Mutillidae: Trogaspidiini) in Northeast Asia highlights its unique distributional pattern. Entomological Science 26(1): e12532. 10.1111/ens.12532

[B11] Pollierer MM Drescher J Potapov A Kasmiatun Mawan A Mutiari M Nazarreta R Hidayat P Buchori D Scheu S (2023) Rainforest conversion to plantations fundamentally alters energy fluxes and functions in canopy arthropod food webs. Ecology Letters 26(10): 1663–1675. 10.1111/ele.14276

[B12] Ramírez MJ (2014) The morphology and phylogeny of dionychan spiders (Araneae: Araneomorphae). Bulletin of the American Museum of Natural History 390: 1–374. 10.1206/821.1

[B13] Ramos D Hartke TR Buchori D Dupérré N Hidayat P Lia M Harms D Scheu S Drescher J (2022) Rainforest conversion to rubber and oil palm reduces abundance, biomass, and diversity of canopy spiders. PeerJ 10: e13898. 10.7717/peerj.13898 PMC9390325 35990898

[B14] Simon E (1895) Descriptions d‘arachnides nouveaux de la famille des Thomisidae. Annales de la Société Entomologique de Belgique 39: 432–443. https://biostor.org/reference/57234

[B15] Simon E (1909) Etude sur les arachnides du Tonkin (1re partie). Bulletin Biologique de la France et de la Belgique 42: 69–147. 10.5962/bhl.part.24151

[B16] Tang G Blick T Ono H (2010) Rediscovery of an obscure spider genus *Zametopina* Simon, 1909 (Araneae, Thomisidae) from Yunnan, China. Bulletin of the National Museum of Nature and Science Tokyo (A) 36: 65–70.

[B17] Thorell T (1877) Studi sui Ragni Malesi e Papuani. I. Ragni di Selebes raccolti nel 1874 dal Dott. O. Beccari. Annali del Museo Civico di Storia Naturale di Genova 10: 341–637. https://biostor.org/reference/72560

[B18] Thorell T (1892) Studi sui ragni Malesi e Papuani. IV, 2. Annali del Museo Civico di Storia Naturale di Genova 31: 1–490. https://biostor.org/reference/104765

[B19] World Spider Catalog (2025) World Spider Catalog. Version 26. Natural History Museum Bern. 10.24436/2 [accessed on 5 August 2025]

